# Supplementary material for: SyNDI: synchronous network data integration framework
Source: BMC Bioinformatics. 2018 Nov 6;19:403. doi: 10.1186/s12859-018-2426-5 (PMC6219086; doi:10.1186/s12859-018-2426-5)
Supplement: Supplementary file 5 — Files of the example presented in Additional file 4. This additional file is a zip package that contains all files used in Additional file 4. Detailed descriptions of each of these files are presented in a README file included in this zip package. (GZ 30411 kb) [file 12859_2018_2426_MOESM5_ESM.gz › SyNDITools/Meme2Fimo/meme_4.9.0_4.tar.gz!/meme_4.9.0/website/html/meme-input.html]

MEME -Introduction


# MEME Suite

The MEME Suite web application requires the use of JavaScript
  
Javascript doesn't seem to be available on your browser.

### MEME -- Multiple EM for Motif Elicitation

### *Motif discovery tool*

---


- **motif distribution**  
  This is where you tell MEME how you believe occurrences of the
  motifs are **distributed**
  among the sequences. Selecting the correct type of distribution
  improves the sensitivity and quality of the motif search.
- **number of motifs**  
  MEME will look for up to this number of distinct motifs in the
  training set. MEME will stop when this number of motifs has been
  found, or when none can be found with *E*-value less than
  10000.
- **number of sites**[optional]  
  This is the total number of sites in the *training set* where
  a single motif occurs. You can choose different limits for the
  **minimum** and **maximum** number of occurrences that MEME
  will consider. If you have prior knowledge about the number of
  occurrences that motifs have in your training set, limiting MEME's
  search in this way can can increase the likelihood of MEME finding
  true motifs.  
  For example, if you know that each motif is likely to occur at
  least 5 times but no more than 8 times in the training set, you
  could specify:

  **Minimum** sites = 5  
  **Maximum** sites = 8

  MEME will then only report motifs with between 5 and 8 occurrences,
  inclusive, in the training set. (The same range [5,...,8] will
  apply, separately, to each motif MEME finds.) MEME may still find
  motifs with slightly fewer or more occurrences then those you
  specify. In the above example, if there is a motif in the training
  set with only 4 occurrences, MEME may still find it, but it will
  report 5 occurrences, one of which will be erroneous. Likewise, if
  a motif in the training set has 9 occurrences, MEME will probably
  still find it, but it will report only 8 of its occurrences.  
  MEME chooses the number of occurrences to report for each motif by
  optimizing a statistical heuristic function, restricting the number
  of occurrences to the range you give here, or using defaults
  described below if you leave these fields blank.  
  **Note 1:**  
  Leave these fields blank if you have selected "**One** per
  sequence" in the "How you think the occurrences... are
  **distributed**" field. In that case, each sequence must have
  exactly one occurrence of each motif. It also doesn't make any
  sense to set the maximum number of sites to a number larger than
  the number of sequences if you have chosen the "**Zero or one**
  per sequence distribution. In that case, there can never be more
  occurrences of a motif than there are sequences.  
  **Note 2:**  
  These fields are optional. If you leave them blank, MEME will
  choose limits depending on the type of occurrence distribution you
  have specified and the number of sequences (*n*) in the
  training set. MEME will also override your settings if they
  conflict with the type of distribution you have chosen (see **Note
  1**, above).

  | Default Numbers of Sites for each Motif | | |
  | --- | --- | --- |
  | type of distribution | minimum sites | maximum sites |
  | **one** occurrence per sequence | *n* | *n* |
  | **zero or one** occurrence per sequence | sqrt(*n*) | *n* |
  | **any number** of repetitions per sequence | sqrt(*n*) | min(5\**n*, 50) |
- **motif width**  
  This is the width (number of characters in the sequence pattern) of
  a single motif. MEME chooses the optimal width of each motif
  individually using a statistical heuristic function. You can choose
  different limits for the **minimum** and **maximum** motif
  widths that MEME will consider. The width of each motif that MEME
  reports will lie within the limits you choose.
    
  **Note on maximum width:**   
  Both protein and DNA motifs are often
  shorter than the default "maximum width" (50).
  It is often advisable for you to reduce that parameter
  to a much smaller value (e.g., in the range 10--20) by
  entering a new value in the box.
- **use a different Markov background model**
- **search given strand only**  
  MEME searches for motifs on both the given DNA strand **and**
  the reverse complement strand by default. Checking this box will
  cause MEME to search the given DNA strand only.
- **discriminative motif discovery**  
  Providing *negative* sequences using this option results in creation of a
  *position-specific prior* (PSP), with the negative sequences and the main (*positive*)
  sequences as inputs to a PSP generator program, which is run as an
  extra step before running MEME. The resulting
  PSP file is used as an additional input to MEME. For more detail,
  see documentation on discriminative motif discovery.
- **look for palindromes only**  
  Checking this box causes MEME to search only for DNA palindromes.
  This causes MEME to average the letter frequencies in corresponding
  motif columns together. For instance, if the width of the motif is
  10, columns 1 and 10, 2 and 9, 3 and 8, etc., are averaged
  together. The averaging combines the frequency of A in one column
  with T in the other, and the frequency of C in one column with G in
  the other. If this box is **not** checked, the columns are
  **not** averaged together.
